# The Psychometric Properties of the Moberg Pick-Up Test (MPUT) to Assess Fine Motor Skills in Adults with Haemophilia

**DOI:** 10.3390/healthcare14030368

**Published:** 2026-01-31

**Authors:** Arnika Lorenz, Fabian Tomschi, Alexander Schmidt, Holger Stephan, Joschua Wiese, Thomas Hilberg

**Affiliations:** Department of Sports Medicine, University of Wuppertal, 42117 Wuppertal, Germany

**Keywords:** hand function, functional test, rare disease, arthropathy, bleeding disorder, Duruöz Hand Index

## Abstract

**Background/Objectives**: Haemophilia-related bleedings primarily affect the musculoskeletal system, and functional tests are used in clinical management. Yet, fine motor skills of the upper extremities have not been evaluated in adult persons with haemophilia (PwH). The Moberg Pick-Up Test (MPUT) assesses fine motor skills but has only been psychometrically evaluated in other cohorts. This study aims to examine its psychometric properties in PwH. **Methods**: A total of 40 moderate or severe PwH A or B were included. The MPUT, consisting of three trials, was conducted twice by rater A and once by rater B. The best performance per hand of each MPUT was used. Subjective hand function (Duruöz Hand Index (DHI) and numeric rating scale (NRS)), elbow joint status (Haemophilia Joint Health Score (HJHS)), pain (NRS), and wrist range of motion (ROM) were utilised for convergent validity evaluation. Inter-rater and test–retest reliability were determined through intraclass correlation coefficients (ICCs) for raw and log10-transformed data. **Results**: Inter-rater and test–retest reliability demonstrated moderate-to-excellent ICCs for both data types (ICC range: 0.624–0.918). The DHI correlated moderately with the average MPUT score of both hands (r = 0.410; *p* = 0.016). Left-hand MPUT scores did not correlate with left elbow HJHS scores, whereas right-hand MPUT scores correlated with right elbow HJHS scores (r = 0.396, *p* = 0.018). Subjective left-hand function (NRS) correlated with the results of the MPUT (r = 0.433; *p* = 0.009). **Conclusions**: The MPUT is a reliable and partially valid tool and can be useful to assess fine motor skills in PwH.

## 1. Introduction

Haemophilia is a rare genetic bleeding disorder, caused by deficient or dysfunctional production of coagulation factors [1], leading to recurrent bleeding episodes primarily affecting the musculoskeletal system [2]. Haemophilia A and B affect approximately 400.000 individuals worldwide, with haemophilia A being more common than haemophilia B [1]. It can be classified based on factor activity into mild (>5%, <40%), moderate (1–5%), and severe (<1%) haemophilia [3]. Repeated haemarthroses can cause structural joint alterations, resulting in haemophilic arthropathy, a chronic condition characterised by, among others, pain [4], a reduced range of motion, and impaired functionality. The ankle, knee, and elbow joints are most commonly affected, and even with modern prophylaxis, haemophilic arthropathy is still a major clinical hallmark [5]. Pain and these structural alterations may restrict daily activities [6], affect psychological well-being [7], reduce subjective physical performance [8], and limit independence, particularly in older people with haemophilia (PwH) [6]. Haemophilic arthropathy is associated with impaired global proprioceptive and neuromuscular performance [9], which may limit functional ability [6].

In clinical practice, the Haemophilia Joint Health Score (HJHS) is one of the most widely used and well-validated tools to assess joint health in PwH [10]. However, the HJHS focuses on joint status and clinical examination findings, including objectively measured range of motion (ROM) as well as assessor-based evaluations of gait and strength, rather than directly assessing functional performance [11]. To address this limitation, the integration of performance-based outcome measures alongside structural and patient-reported assessments has been advocated [12,13]. Moreover, there is a need for sensitive tools to evaluate functional ability and musculoskeletal performance in PwH [10,14]. Functional performance tests can provide objective, quantifiable data on functional ability and mobility safety and may detect subtle changes in performance of PwH [15,16]. Common performance-based tests such as the Timed Up and Go (TUG), Single Leg Stance (SLS), and Six-Minute Walk Test have been used to evaluate lower limb function, balance, and endurance in PwH [13]. For example, the excellent reliability and validity of the TUG test was recently demonstrated in adults with haemophilia, confirming the feasibility of performance-based testing in this population [17]. Despite these advances in performance testing, there is currently no valid test available to assess functional performance of the upper extremities, although limitations in elbow mobility often occur [18] and substantially affect hand dexterity, which is essential for daily activities and independence [19]. However, psychometric evaluations of upper-limb performance tests remain underexplored in this population [12]. While the HJHS provides important information on joint status and impairment, and the above-described functional tests focus on lower limb function, a short performance-based assessment such as the Moberg Pick-Up Test (MPUT) may provide complementary information on fine motor skills in PwH.

The MPUT is a standardised, time-based assessment of fine motor skills of the upper extremities. Originally, it was developed by Moberg (1958) [20] and subsequently modified by Dellon (1981) [21]. It has demonstrated strong reliability and validity in populations with hand impairments including osteoarthritis [22], rheumatoid arthritis [23], and carpal tunnel syndrome [24]. Given the strong association between hand function, autonomy [25], and quality of life [26], evaluating fine motor skills represents an important yet understudied aspect of functional assessment in PwH. Further, impaired fine motor skills are associated with an increased risk of mortality [27] and a slower walking speed [27,28]. As fine motor skills assessed by the MPUT are essential for daily activities, the test not only reflects joint structure but may provide valuable complementary information to joint-based measures in PwH [29].

Based on these considerations, the present study aims to evaluate the psychometric properties, specifically the reliability and convergent validity, of the MPUT in adults with haemophilia A or B. Test–retest reliability, measurement error, minimal detectable change (MDC), and convergent validity are to be determined in relation to outcomes such as the HJHS and patient-reported Duruöz Hand Index (DHI).

Accordingly, the following hypotheses are formulated: (1) The MPUT shows a high test–retest and inter-rater reliability. (2) The MPUT demonstrates convergent validity in PwH, with a moderate-to-strong correlation with the DHI and HJHS.

## 2. Materials and Methods

### 2.1. Study Design

The study was conducted in accordance with the Declaration of Helsinki. Written informed consent was obtained from all participants, and the study protocol was approved by the local ethics committee of the University of Wuppertal (Wuppertal, Germany) (reference: SK/AE 250411).

For reliability assessment, the MPUT was administered twice by rater A (A.L.) to determine test–retest reliability and once by rater B (F.T.) to evaluate inter-rater reliability with approximately 60 min between assessments as previously described [17,30]. All measurements were performed by researchers experienced in conducting functional assessments in PwH. The testing order of the MPUT was randomised by an independent researcher, who was not otherwise involved in the study. All additional assessments were performed by experienced researchers.

For evaluation of the convergent validity, all participants were assessed once following a standardised protocol. These assessments included the examination of hand fine motor skills using MPUT, the DHI, and subjective hand function on a numeric rating scale (NRS). Additionally, the orthopaedic joint status using the Haemophilia Joint Health Score (HJHS), and the range of motion (ROM) for hand flexion/extension as well as radial/ulnar deviation of wrist joints, and the individual pain intensity on an NRS were assessed. Demographic and clinical data, including age, height, mass, body mass index (BMI), handedness, haemophilia type and severity, and the annual bleeding rate (ABR) were recorded as patient characteristics.

Because the MPUT has not previously been investigated in PwH, comparator measures for convergent validity were selected to reflect different but related aspects of fine motor performance. Accordingly, these included joint status, perceived hand-related activity limitations, wrist mobility, pain, and subjective hand function. The HJHS was used as a well-established disease-specific measure of joint status in haemophilia, and the DHI, although primarily used in rheumatology and other musculoskeletal populations, was included as a patient-reported measure reflecting perceived hand-related limitations.

### 2.2. Participants and Sample Size Calculations

For evaluation of inter-rater reliability and test–retest reliability, the required sample size was calculated using Walter’s formula [31]. The calculation was based on the study of Amirjani et al. [24], which evaluated the test–retest reliability in patients with carpal tunnel syndrome. Based on an intraclass correlation coefficient (ICC) of 0.91, a power of 0.80 and an alpha level of 0.05, a minimum total sample size of n = 30 was required. Due to the testing conditions, a dropout rate of 10% was assumed, therefore n = 33 participants were needed.

The required sample size to determine validity was calculated using G*Power 3.1.9.7 software (Heinrich Heine University Düsseldorf, Düsseldorf, Germany). The calculation was based on a medium correlation (r = 0.4) between MPUT and grip strength in patients with hand osteoarthritis, as reported by Silva et al. (2017) [22]. As no haemophilia-specific reference data were available, this effect size was used as the closest available clinical comparator. Using an alpha error of 0.05 and a power of 0.80, the analysis indicated that a minimum of 34 PwH was needed. Due to the testing conditions and the expected response rate of the questionnaires, a dropout rate of 15% was assumed, which was higher than in the reliability analysis for that reason. This resulted in a required sample size of 40 PwH.

Adult persons suffering from moderate or severe haemophilia A or B were eligible for inclusion. Persons with other bleeding disorders, a history of elbow surgery (including, but not limited to, radiosynoviorthesis) within six months prior to assessment, or recent bleeding of the upper extremities within 10 days prior to assessment were excluded [14]. Other exclusion criteria were different rheumatologic diseases such as ankylosing spondylitis, psoriasis, or other local or generalised joint infections (e.g., borreliosis, septic arthritis), (diabetic) neuropathy, or other neurological disorders. Participants were advised to wear glasses if necessary.

### 2.3. MPUT Procedure

To assess fine motor skills, the MPUT was performed according to a standard protocol with open eyes only [32]. Conducting the MPUT with closed eyes would likely increase the sensory demands of the task, as visual feedback is missing. In line with the aim of the study to evaluate upper-limb motor function, particularly fine motor skills in PwH, the MPUT was therefore administered exclusively with open eyes to avoid such confounding effects.

PwH were seated in front of a table, where twelve metallic objects were placed randomly 15 cm from the edge of the table in a square measuring 15 cm, whereas no specific surface was used. A small box with a diameter of 8 cm and a height of 4 cm was placed in front of the participants. The objects to pick up included one wing nut, screw, key, nail, Canadian nickel, Canadian quarter, washer, safety pin, paper clip, large- and medium-sized hexagonal nuts, and small square nut. PwH were instructed to place the metallic items separately with their fingertips without using the fourth and fifth digits and place them separately in the box as quickly as possible. They were advised not to slide the objects to the edge of the table and pick them up there. For instruction, standardised wording was used. The time to completion was recorded using a stopwatch with a precision of 1/100 s [28]. The examiner asked whether the participant was ready, and timing began after a three second countdown and stopped when the participant completed the task. The starting hand was randomised by another independent researcher, giving equal probability to the dominant and non-dominant hand. The dominant hand was defined as the one used for writing. Each MPUT consisted of three trials per hand, and each hand was used alternately starting with one hand following the randomisation list. The best performance of the three trials per hand was used for analysis [33].

### 2.4. Additional Assessments

Subjective hand function was assessed using German-translated DHI, a validated and standardised self-report questionnaire evaluating limitations of the hand function (score range 0–90, with higher scores indicating greater limitations). DHI consists of five subscales: in the kitchen, dressing, hygiene, in the office, and other [34]. Subjective hand function of each hand was additionally assessed using an NRS. Participants rated their hand function on a 0–10 scale, with higher values indicating better functional ability. Subjective average pain levels in the elbows, shoulders, and wrists over the past two weeks were assessed using NRS (0–10 scale, with higher values indicating greater pain intensity) [35].

To assess the clinical joint situation, the HJHS was used, which evaluates swelling, muscle atrophy, crepitus on motion, axial deformity, and ROM. Higher score points indicate greater functional and structural joint impairment as a sign of more pronounced haemophilic arthropathy [11,36]. Additionally, wrist ROM for flexion/extension as well as radial/ulnar deviation were measured using a universal goniometer.

### 2.5. Statistics

For statistical analysis IBM SPSS Statistics (Version 29.0.2; IBM Corp., Armonk, NY, USA) was used. Normal distribution was tested using the Shapiro–Wilk test. Shapiro–Wilk test revealed non-normal distributions, therefore log10-transformed data were used to achieve a normal-like distribution. Inter-rater and test–retest reliability were determined through ICCs. Analyses were performed on both raw and base-10 logarithmically transformed data (log10-transformed) to check the plausibility of the results based on non-transformed data.

Because of the robustness of ANOVA-based ICC analyses, original non-transformed data were primarily used, unless otherwise specified [37]. For subsequent analyses and visualisation (e.g., Bland–Altman Plots, standard error of measurement (SEM), and MDC) were used to facilitate the interpretation.

Specifically, the ICC (3,1) two-way mixed-effects model with absolute agreement for single measurements was used [38]. ICC values were reported with 95% confidence intervals and interpreted according to the following guidelines by Koo and Li (2016): <0.50 = poor, 0.50–0.75 = moderate, 0.75–0.90 = good, and >0.90 = excellent reliability [39].

For visual representation of agreements, Bland–Altman plots of non-transformed data were created using Microsoft Excel (Version 2511, Microsoft 365; Microsoft, Redmond, WA, USA). The standard error of measurement (SEM) was calculated using the formula: SEM = SD_pooled_ × $\sqrt{1-ICC}$, where SD represents the pooled standard deviation and ICC refers to the non-transformed test–retest calculation. The following formula was used to calculate the MDC: MDC_95_ = 1.96 × $\sqrt{2}$ × SEM.

For convergent validity, Spearman’s rank correlation coefficients between the average MPUT scores of both hands and DHI were calculated. Further, correlation coefficients between best MPUT performance and ROM of the hands, subjective hand function, and joint condition of the elbow were determined by using Spearman’s rank correlation coefficients. Correlation coefficients were interpreted as follows: 0.1 < r < 0.3 = small, 0.3 < r < 0.5 = moderate, and r > 0.5 = large effect size [40].

## 3. Results

### 3.1. Participants

In total, 40 PwH were included in the validity analyses, of whom 33 were also included in the reliability analyses according to the a priori sample size calculation. Two participants were excluded due to other blood coagulation disorders or polyneuropathies.

Questionnaires were missing for two participants, who were excluded for validity analyses. In addition, one participant had a left arm deformity, and another had an amputated right thumb, so these limbs were excluded. Consequently, data from 36 PwH (age: 53.0 (45.5, 60.5)) were used for validity analyses. In detail, average MPUT data from 34 PwH and hand-specific data from 70 hands were available.

For reliability analyses, data from 31 PwH (age: 56.0 (47.5, 61.0) were available. Considering the individual hand characteristics described above, MPUT data from a total of 60 hands were analysed for reliability.

Table 1 presents anthropometric and disease-specific data of PwH. All PwH received prophylactic therapy except for one who received on-demand therapy. No persons with mild haemophilia were included.

### 3.2. Reliability

In Table 2, the MPUT results of 30 PwH for each hand under all three testing conditions are presented. As the data were not normally distributed, both median and mean MPUT values are reported.

The ICC calculations demonstrated good-to-excellent test–retest reliability. Inter-rater reliability ranged from moderate to good depending on the different testing conditions. For instance, test–retest reliability of the right-hand MPUT was good (ICC = 0.829, 95% CI = 0.672–0.915). The SEM (1.07 s) and MDC_95_ (2.97 s; 23.3% of the mean performance time) were calculated from non-transformed data. All ICC values for both log10-transformed and non-transformed data are presented in Table 3. The ICCs from both data types were comparable across all testing conditions, and the interpretation did not differ.

**Figure 1:**
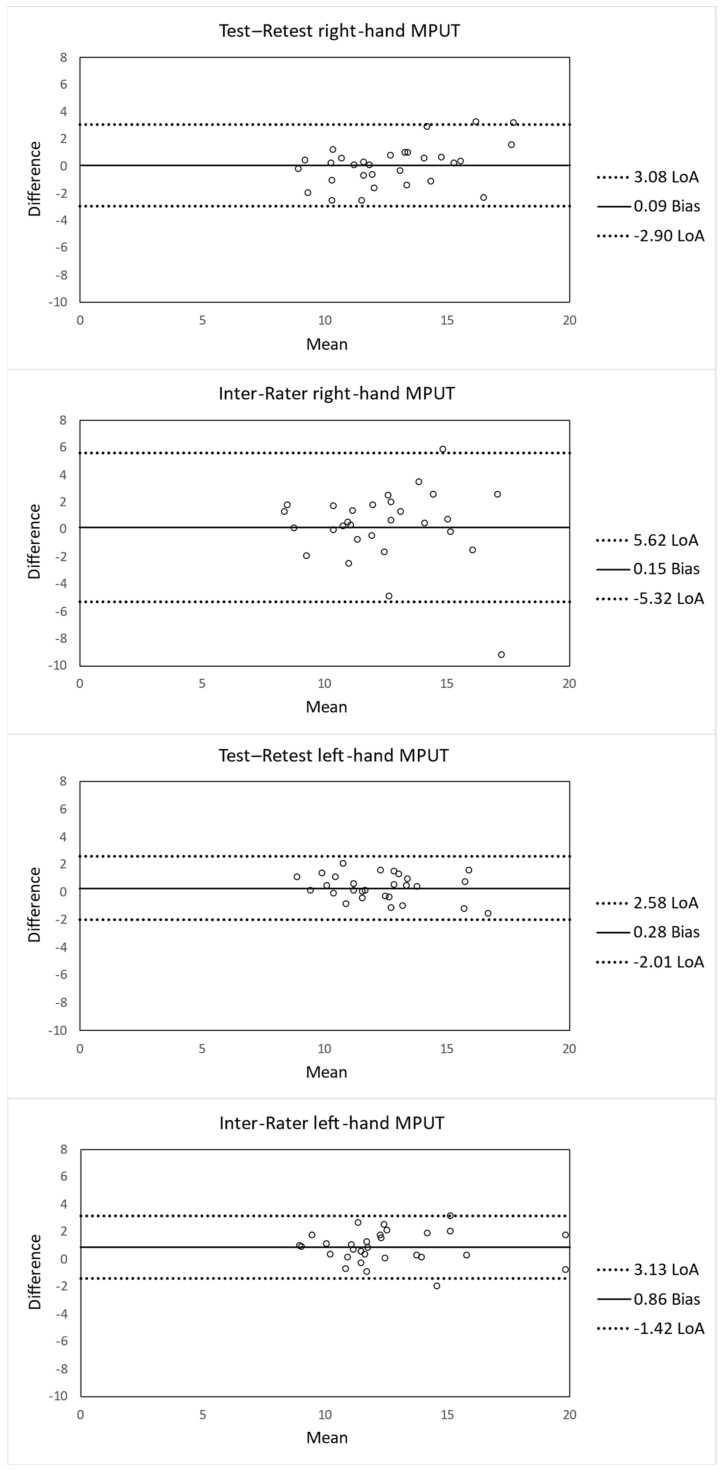
Bland–Altman plots with the mean difference (Bias) and 95% upper and lower limits of agreement (LoA) for test–retest and inter-rater measurements of the MPUT for both hands.

The Bland–Altman analyses revealed minimal systematic bias across all testing conditions. The test–retest measurements demonstrated narrow limits of agreement (LoA), whereas the inter-rater analyses showed greater dispersion, particularly for the right hand. Mean differences (biases) and 95% LoA for test–retest and inter-rater reliability are displayed as Bland–Altman plots in Figure 1.

### 3.3. Validity

Convergent validity of the MPUT was assessed by computing correlations with disease-specific and functional measures. The average MPUT score of both hands showed significant moderate correlations with the DHI total score (r = 0.410, *p* = 0.016), as well as with the DHI “dressing” (r = 0.360, *p* = 0.037) and the DHI “other” (r = 0.417, *p* = 0.014) (Table 4). There was no significant correlation between the MPUT and HJHS of both elbows (r = 0.296, *p* = 0.089).

Right-hand MPUT performance demonstrated moderate correlations with the DHI total score (r = 0.352, *p* = 0.038), the HJHS of the right elbow (r = 0.396, *p* = 0.018), and the wrist flexion/extension range of motion (r = 0.463, *p* = 0.005). Left-hand MPUT scores showed moderate correlations with the DHI total score (r = 0.439, *p* = 0.008), left-hand function (r = 0.433, *p* = 0.009), and pain in the left shoulder (r = 0.369, *p* = 0.029) (Table 5).

## 4. Discussion

In this study, the psychometric properties of the MPUT were examined in adults with haemophilia A or B, specifically its reliability and validity, as well as the measurement error and minimal detectable change. The main results indicate that the MPUT is a reliable assessment tool for upper extremity fine motor skills with good-to-excellent test–retest reliability and moderate-to-good inter-rater reliability in PwH, thus confirming the hypothesis (1). The results further indicate evidence for the convergent validity of the MPUT, with moderate correlations found between the MPUT and especially DHI scores, while correlations with other constructs were low and not consistent. Hence, Hypothesis (2) can only be partially confirmed.

In more detail, the results dealing with reliability analyses reveal good test–retest reliability for the right hand and excellent test–retest reliability for the left hand. Inter-rater reliability for the left hand was good, whereas the right hand showed only a moderate inter-rater reliability (Table 3). The biases for all testing conditions were close to zero (0.09–0.86), indicating stable performances between measurements. The LoA showed greater dispersion, especially for the right hand in the inter-rater comparison, but the variability can still be considered acceptable for performance tests [41,42].

Based on these results, the MPUT scores seem to be more stable when assessed by the same rater, although a standardised instruction protocol was used. This reflects a common phenomenon, likely caused by differences in training and experience [43]. Therefore, in clinical practice, it is recommended that the test be performed by the same rater for each patient. In addition, the inter-rater agreement might benefit from increased rater experience with the MPUT, even when a standardised instruction protocol is used. Furthermore, the lower reliability and wide LoA for the right hand might be caused by handedness in combination with elbow arthropathy and test familiarisation.

Compared to the study by Amirjani et al. (2011), which examined the test–retest reliability of the mean MPUT score in patients with carpal tunnel syndrome (ICC = 0.91), the test–retest reliability observed in the present study was similar for the left hand but lower for the right hand [24]. The described study included both women and men, whereas the present study investigated reliability only in male participants. Moreover, carpal tunnel syndrome primarily affects the hands, whereas the elbows are more frequently affected in PwH. Ng et al. (1999) observed a good inter-rater reliability of the MPUT in a small sample of healthy subjects [32]. However, these correlation coefficients cannot be directly compared to the ICCs in the present study, as they describe linear associations rather than absolute agreement.

Herein, the results regarding the convergent validity of the MPUT are presented. It is important to consider that there is currently no gold standard for assessing fine motor skills of the upper extremities. Therefore, different subjective and objective assessment tools were used to examine correlations with the MPUT for convergent validity testing. These analyses showed that the average MPUT scores of both hands correlated moderately with the DHI total score and the subscale “other” (Table 4). The right-hand MPUT showed moderate correlations with the DHI, the HJHS of the right elbow, and the flexion/extension ROM of the wrist. However, no correlation with subjective right-hand function, assessed using an NRS, was found.

In contrast, the left-hand MPUT did not correlate with the HJHS of the left elbow or the ROM but did correlate with the DHI and subjective left-hand function (Table 5). As 91.7% of PwH were right-handed in the present study, these results suggest that the fine motor skill impairment is associated with elbow arthropathy of the dominant (right) side, but not with the non-dominant side. This interpretation is further supported by the absence of a significant correlation between the average MPUT score of both hands with the HJHS scores for both elbows. However, the small number of left-handed participants may limit definitive conclusions regarding lateralised effects.

Silva et al. (2017) found a correlation between the MPUT and pain intensity in women with osteoarthritis, whereas the present study did not observe any correlation to pain of the wrists [22]. This discrepancy may be explained by the fact that the hands are less frequently affected in PwH compared to the patients in the study of Silva et al. (2017) [5,22]. However, in the present study, subjective hand function, assessed by the DHI, showed moderate correlations with the MPUT scores for both hands separately as well as with the average score of both hands. Therefore, the results support convergent validity, indicating that worse subjective hand function, reflected by higher DHI total scores, is associated with longer completion times in the MPUT. Overall, these findings further suggest that the currently available objective and valid measurement tools may not fully capture all aspects of fine motor skills in PwH, highlighting the potential usefulness of the MPUT. While measures such as the HJHS and ROM primarily assess joint status (structural changes, pain, or movement restrictions), they do not directly reflect fine motor skills. Importantly, fine motor skills may be reduced even if joint scores or ROM appear normal, as individuals may compensate to maintain function. This suggests that interventions addressing not only joint mobility but also hand–eye coordination and precision grip may be required to increase fine motor skills [29,44]. Therefore, the MPUT may provide complementary information on upper limb function beyond established measures of joint status such as the HJHS and performance-based tests primarily targeting function of lower limbs.

Mean MPUT scores for the right and left hand under the testing conditions ranged from 12.1 to 13.0 s (median: 11.4 to 12.7 s), with an SEM of 1.07 s and an MDC_95_ of 2.97 s. These scores suggest that PwH completed the MPUT faster than healthy men in the study by Santos-Eggimann et al. (2020) [28], who reported a mean score of 13.1 s for the dominant hand. However, the median age of PwH in the present study was 54 years, whereas the participants in the described study were older with a mean age of 73.4 years [28]. Moreover the differences were smaller than the SEM. Additionally, Ng et al. (1999) reported MPUT scores of 11.5 to 11.9 s in healthy younger subjects [32], and a more recent study showed that hand function decreases within older age [33]. Further, Silva et al. (2017) [22] demonstrated significant differences in MPUT performance using another protocol between women with hand osteoarthritis (right hand: 23.3 s; left hand: 22.5 s) and healthy women with a mean age of 64.5 years (right hand: 17.3 s; left hand: 18.4 s).

The present study provides the first insights into the psychometric characteristics of the MPUT in PwH. However, future studies are necessary to investigate differences in fine motor skills between PwH and healthy controls. Potential influencing factors, such as age, should also be analysed to further examine the convergent validity in PwH. In addition, previous studies have shown that hand function is closely linked to autonomy and quality of life [25,26], and that impaired fine motor skills are associated with an increased risk of mortality [27] and a slower walking speed [27,28]. Examining the association between MPUT performance and these factors in PwH may be of particular interest for future research, especially to establish potential cutoff values for the MPUT.

## 5. Strengths and Limitations

To the best of our knowledge, this is the first study to evaluate the psychometric properties of the MPUT to assess fine motor skills in PwH. Despite the broad range in age and weight, as well as the differences in treatment regimens, reliability and partial validity of the MPUT could be demonstrated. The required sample size was calculated a priori for both reliability as well as validity testing. Given that haemophilia is a rare disease, this study included a sufficient number of PwH.

Yet, some potential limitations must be acknowledged to adequately interpret the results obtained. First, it must be considered that there is no assessment that is considered to be the gold standard in fine motor skill testing of the upper extremities. Therefore, specific subjective and objective assessments were selected in this study to test the convergent validity of the MPUT. It must be acknowledged that the validity might have also been examined using other established questionnaires such as, for instance, the Haemophilia Activity List questionnaire (HAL; dimension “Function of the arms”) [45]. Comparisons with the Quick Disability of the Arm, Shoulder and Hand scale (Quick DASH) [46], or the physical component of the disability dimension in the Health Assessment Questionnaire (HAQ) [47], both valid and reliable measures in persons with rheumatoid arthritis, may have also been valuable in this context. Additionally, the sample size was calculated based on an estimated effect size of r = 0.4, derived from previously reported correlations between the MPUT and grip strength as the literature in this context is rather scarce. As the correlations between the MPUT and other assessments (DHI, partially ROM, and partially HJHS) may have been smaller in this study, the a priori calculation might have slightly underestimated the required sample size.

As this study included only adult male participants with moderate-to-severe haemophilia A or B, who were mostly right-handed and receiving prophylactic therapy, the findings may not be generalisable to individuals with mild haemophilia, female PwH, children with haemophilia, or patients receiving on-demand therapy or who are left-handed. Future studies should therefore include more diverse populations to assess the MPUT across disease severities, sexes, and age groups.

## 6. Conclusions

The present study provides a basis for further use of the MPUT to evaluate the functional performance of fine motor skills in PwH in a clinical context. The results demonstrated moderate-to-excellent reliability, with higher ICCs observed for test–retest reliability compared to inter-rater reliability. Evidence for the convergent validity of the MPUT was found, as the MPUT showed significant correlations with subjective measures of hand function (DHI), while other measures were not correlated. Considering these findings, the MPUT seems to be a useful, quick, feasible, and safe performance-based test to evaluate fine motor skills as part of upper-limb function, particularly when joint-based measures may not fully capture functional deficits.

## Figures and Tables

**Table 1:** Anthropometric and disease-specific data of PwH. Data are presented as median (Q1, Q3), unless otherwise specified.

	PwH, Validity (n = 36)	PwH, Reliability (n = 31)
Parameter	Median (Q1, Q3)	Median (Q1, Q3)
**Age [years]**	53.0 (45.5, 60.5)	56.0 (47.5, 61.0)
**Mass [kg]**	80.0 (75.5, 85.3)]	80.0 (76.5, 85.3)
**Height [cm]**	176.5 (172.5, 183.0)	176.0 (173.0, 181.0)
**Body Mass Index [kg/m^2^]**	25.3 (23.5, 27.1)	25.4 (23.6, 27.1)
**HJHS total [points]**	25.0 (11.0, 45.5)	31.0 (11.5, 47.5)
**HJHS right elbow [points]**	2.0 (0.0, 8.0)	2.0 (0.0, 8.5)
**HJHS left elbow [points]**	4.0 (1.0, 7.0)	5.0 (1.0, 7.0)
**ABR**	0.5 (0.0, 2.0)	1.0 (0.0, 1.5)
**Dominant hand [n (%)]**	Right: 33 (91.7%) Left: 3 (8.3%)	Right: 28 (90.3%) Left: 3 (9.7%)
**Haemophilia type [n (%)]**	A: 28 (77.8%) B: 8 (22.2%)	A: 23 (74.2%) B: 8 (25.8%)
**Haemophilia severity [n (%)]**	Severe: 31 (86.1%) Moderate: 5 (13.9%)	Severe: 26 (83.9%) Moderate: 5 (16.1%)

Abbreviations: ABR, annual bleeding rate; HJHS, Haemophilia Joint Health Score; PwH, persons with haemophilia.

**Table 2:** Moberg Pick-Up Test (MPUT) values under three different testing conditions. Data are presented as mean ± standard deviation (SD) and median (Q1, Q3).

n = 30 per Hand	Testing Condition		
	Rater A, Test 1	Rater A, Test 2	Rater B, Test 1
**Right-hand MPUT [s]**			
Mean ± SD	12.8 ± 2.9	12.7 ± 2.2	12.6 ± 3.5
Median (Q1, Q3)	12.2 (10.9, 15.1)	12.7 (11.1, 14.4)	12.0 (10.6, 13.9)
**Left-hand MPUT [s]**			
Mean ± SD	13.0 ± 2.7	12.7 ± 3.1	12.1 ± 2.7
Median (Q1, Q3)	12.4 (11.3, 13.9)	12.2 (10.9, 13.6)	11.4 (10.8, 13.6)

**Table 3:** Test–retest and inter-rater reliability of Moberg Pick-Up Test (MPUT) presented as Intraclass Correlation Coefficients (ICCs) with 95% confidence interval (CI).

n = 30 per Hand	Test–Retest (Rater A)	Inter-Rater (Rater A–Rater B)
**Right-hand MPUT**		
ICC non-transformed data (95% CI)	0.829 (95% CI: 0.672–0.915)	0.624 (95% CI: 0.342–0.802)
ICC log10-transformed data (95% CI)	0.837 (95% CI: 0.685–0.919)	0.667 (95% CI: 0.408–0.826)
**Left-hand MPUT**		
ICC non-transformed data (95% CI)	0.918 (95% CI: 0.836–0.960)	0.867 (95% CI: 0.574–0.948)
ICC log10-transformed data (95% CI)	0.914 (95% CI: 0.822–0.959)	0.848 (95% CI: 0.489–0.942)

Abbreviations: log10-transformed, base-10 logarithmically transformed.

**Table 4:** Spearman correlation coefficients between average Moberg Pick-Up Test (MPUT) score of both hands with total score of Duruöz Hand Index (DHI) and its subscales, as well as Haemophilia Joint Health Score (HJHS) of both elbows. Data are presented as r_s_ values with corresponding *p*-values. Significant (*p* < 0.05) correlations are shown in bold.

n = 34 PwH	DHI– Total	DHI–Kitchen	DHI–Dressing	DHI–Hygiene	DHI–Office	DHI–Other	HJHS Both Elbows
**Mean MPUT score (both hands)**	**0.410**	0.322	**0.360**	0.146	0.233	**0.417**	0.296
***p*** **-value**	0.016	0.064	0.037	0.411	0.184	0.014	0.089

**Table 5:** Spearman correlation coefficients between the Moberg Pick-Up Test (MPUT) score for both hands with total score of Duruöz Hand Index (DHI), Haemophilia Joint Health Score (HJHS) of each elbow, range of motion (ROM) in flexion and extension, ulnar and radial deviations of wrists, and subjective hand function assessed by a numeric rating scale (NRS). Data are presented as r_s_ values with corresponding *p*-values. Significant (*p* < 0.05) correlations are shown in bold.

n = 35 per Hand	DHI–Total	Pain Elbow	Pain Shoulder	Pain Wrist	HJHS Elbow	Wrist ROM Flexion/Extension	Wrist ROM Radial/Ulnar	Hand Function
**Right-hand MPUT**	**0.352**	0.096	0.045	0.127	**0.396**	**0.463**	0.057	0.264
***p*-value**	0.038	0.584	0.797	0.467	0.018	0.005	0.743	0.125
**Left-hand MPUT**	**0.439**	0.128	**0.369**	0.149	0.061	0.307	0.075	**0.433**
***p*-value**	0.008	0.465	0.029	0.394	0.728	0.073	0.669	0.009

## Data Availability

The data presented in this study are available on request from the corresponding author due to ethical and privacy considerations.

## Abbreviations

The following abbreviations are used in this manuscript:

ABR	annual bleeding rate
Bias	mean difference
CI	confidence interval
DASH	Disability of the Arm, Shoulder and Hand scale
DHI	Duruöz Hand Index
HAL	Haemophilia Activity List
HAQ	Health Assessment Questionnaire
HJHS	Haemophilia Joint Health Score
ICC	intraclass correlation coefficient
LoA	limits of agreement
log10-transformed	base-10 logarithmically transformed
MDC	minimal detectable change
MPUT	Moberg Pick-Up Test
NRS	numeric rating scale
ROM	range of motion
PwH	persons with haemophilia
SEM	standard error of measurement
SLS	Single Leg Stance
TUG	Timed Up and Go
